# Genome-wide association analysis on pre-harvest sprouting resistance and grain color in U.S. winter wheat

**DOI:** 10.1186/s12864-016-3148-6

**Published:** 2016-10-12

**Authors:** Meng Lin, Dadong Zhang, Shubing Liu, Guorong Zhang, Jianming Yu, Allan K. Fritz, Guihua Bai

**Affiliations:** 1 Agronomy Department, Kansas State University, Manhattan, KS 66506 USA; 2 Agronomy Department, Iowa State University Ames, Iowa, 50011 USA; 3 Hard Winter Wheat Genetics Research Unit, USDA-ARS, Manhattan, KS 66506 USA

**Keywords:** *Triticum aestivum*, pre-harvest sprouting resistance, grain color, genome- wide association studies

## Abstract

**Background:**

Pre-harvest sprouting (PHS) in wheat can cause substantial reduction in grain yield and end-use quality. Grain color (GC) together with other components affect PHS resistance. Several quantitative trait loci (QTL) have been reported for PHS resistance, and two of them on chromosome 3AS (*TaPHS1*) and 4A have been cloned.

**Methods:**

To determine genetic architecture of PHS and GC and genetic relationships of the two traits, a genome-wide association study (GWAS) was conducted by evaluating a panel of 185 U.S. elite breeding lines and cultivars for sprouting rates of wheat spikes and GC in both greenhouse and field experiments. The panel was genotyped using the wheat 9K and 90K single nucleotide polymorphism (SNP) arrays.

**Results:**

Four QTL for GC on four chromosomes and 12 QTL for PHS resistance on 10 chromosomes were identified in at least two experiments. QTL for PHS resistance showed varied effects under different environments, and those on chromosomes 3AS, 3AL, 3B, 4AL and 7A were the more frequently identified QTL. The common QTL for GC and PHS resistance were identified on the long arms of the chromosome 3A and 3D.

**Conclusions:**

Wheat grain color is regulated by the three known genes on group 3 chromosomes and additional genes from other chromosomes. These grain color genes showed significant effects on PHS resistance in some environments. However, several other QTL that did not affect grain color also played a significant role on PHS resistance. Therefore, it is possible to breed PHS-resistant white wheat by pyramiding these non-color related QTL.

**Electronic supplementary material:**

The online version of this article (doi:10.1186/s12864-016-3148-6) contains supplementary material, which is available to authorized users.

## Background

Pre-harvest sprouting (PHS) of wheat (*Triticum aestivum* L.) refers to the germination of wheat grains in matured spikes before harvest due to continuous wet weather during harvest seasons. PHS can result in a significant reduction in wheat grain yield and grain end-use quality, thus a reduction in grain sale price [1, 2]. Growing PHS-resistant cultivars is the most effective way to minimize PHS damage. PHS resistance QTL have been reported on almost all wheat chromosomes. One major QTL mapped on chromosome 3AS, designated as *TaPHS1*, has been cloned [3, 4]. Another major QTL on chromosome 4AL has been fine mapped with single nucleotide polymorphisms (SNPs) [5–7]. Recently, several candidate genes have been reported for the 4A QTL in different studies [6, 8]. In addition, several minor QTL have also been reported on chromosomes 2B [9–12], 3D [13], 4B, 4D [14] and many others [15].

Wheat grain color (GC) has long been associated with PHS, and red-grained wheats are usually more tolerant to PHS than the white-grained wheats [16–18]. The pigments, catechin and proanthocyanidins (PAs) synthesized through the flavonoid synthesis pathway, result in red GC [19, 20]. Early cytogenetic studies suggested that three genes, *R-A1*, *R-B1* and *R-D1*, on homoeologous group 3 chromosomes control GC [21–23], and show a pleiotropic effect on wheat PHS resistance by accumulating catechin, a precursor of the red pigment, that inhibits grain germination [19, 24]. Flinthman [16] found that grain dormancy levels were increased in white-grained wheat NS-67 after adding a single GC (*R*) gene to one of group 3 chromosomes. Groos et al. [1] identified common QTL for GC and PHS resistance on chromosomes 3AL, 3BL, 3DL and 5A in a white × red wheat cross. The white-grained mutants of 'Chinese Spring' and 'AUS1490' showed increased sprouting, indicating that *R* genes enhanced PHS tolerance [17, 18]. Recently, *Tamyb10* genes, the transcription factors of the flavonoid biosynthetic pathway, have been reported as candidate genes for the GC trait [25]. However, how much these *R* genes contribute to PHS resistance remains unknown. Therefore, simultaneous genome-wide association studies (GWAS) on both traits may reveal the relationship between *R* genes and PHS resistance.

Genome-wide association studies have been conducted in many plant species to discover and validate QTL and genes for various traits. By taking advantages of historical recombination events and linkage disequilibrium (LD) between causal genetic variants and nearby SNPs, GWAS detects statistical associations between genetic variations and phenotypic variations throughout the genome [26–29]. Therefore, GWAS can potentially increase mapping resolution by taking advantages of historical recombinations using highly diverse populations. To date, GWAS has not been reported for GC, and only several studies have been reported for wheat PHS resistance [11, 30–32]. In the current study, we analyzed a panel of elite breeding lines and cultivars from major U.S. winter wheat breeding programs using the wheat 9K and 90K arrays to (1) study the phenotypic variance of PHS resistance in U.S. winter wheat, (2) identify genome-wide QTL for GC and PHS resistance, and (3) determine the genetic relationship between GC and PHS resistance.

## Methods

### Plant materials

A set of 185 winter wheat accessions [33] was assembled to include 130 hard winter wheat and 55 soft winter wheat accessions as listed in Additional file 1: Table S1. A mapping population of 155 F_6_ recombinant inbred lines (RILs) derived from the cross of Tutoumai A x Siyang 936 [7, 34] was used to validate the SNPs that showed significant associations with the *Qphs.hwwgr-4A*.

### Pre-harvest sprouting evaluation

In the greenhouse experiments, five plants per accession were grown in a 13 by 13 cm Dura-pot (Hummert Int. Topeka, KS) under the growth condition listed in Additional file 2: Table S2 after vernalization for 7 weeks at 4 °C in a cold chamber. The GWAS panel was evaluated for PHS in the greenhouse experiments of fall (August-December) 2011, spring (January-May) and fall 2012, and spring 2013. All experiments were conducted in a randomized complete block design with two replications of five plants.

The GWAS panel was also planted for PHS resistance evaluation in the Kansas State University Rocky Ford Wheat Research Farm, Manhattan, KS and the Agricultural Research Center-Hays, Hays, KS, respectively, in the summers of 2013 and 2014. About 30 seeds per accession were planted in a 1.22-m-long single-row plot, and each experiment had two replications.

When wheat plants reached physiological maturity, similar to Zadoks scale 91 [35], spikes that lost green color [36] were harvested from both greenhouse and field experiments, and evaluated for PHS in the lab. Five spikes per accession that were harvested from each replicate were air-dried for 5 d in a greenhouse, and then stored at -20 °C to maintain dormancy for PHS evaluation. After all accessions had been collected, the greenhouse- harvested spikes were air-dried 9 d and field-harvested spikes for 5 d at room temperature. The additional drying days were determined based on preliminary test results of randomly selected samples from field and greenhouse experiments that maximize phenotypic differences among genotypes. After the dried spikes had been immersed in de-ionized water for 12 h, they were enclosed in a moist chamber at 22 ± 1 °C with an attached humidifier that ran twice daily at 2 h each time to maintain high moisture in the chamber. After 7 d of incubation, the germinated and non-germinated kernels were hand-threshed and counted separately to calculate the percentage of germinated kernels from all five spikes of each replication.

### Evaluation of grain color

Grain color was evaluated for grains harvested from one field experiment (2009-2010 Enid Oklahoma) and the fall 2011 greenhouse experiment at Manhattan KS. For each accession, ten seeds were soaked in 1 M sodium hydroxide (NaOH) for 1 h to increase the color contrast. Grain color intensity was determined visually using a scale of 1 to 4, where 1 represents white, 2 light red, 3 red and 4 dark red.

### DNA isolation and genotyping

Leaf tissue was collected at the two-leaf stage, and genomic DNA was isolated using a modified cetyltrimethyl ammonium bromide (CTAB) method [33]. A total of 446 polymorphic SSR markers were selected to genotype the association panel based on PCR product quality, chromosome distribution in available genetic maps (http://wheat.pw.usda.gov/GG3/; verified 11 Aug. 2010), and previously reported associations with PHS resistance. One expression sequence tag (EST), *ZXQ118* [12] and three gene markers of *PM19A1* and *PM19A2* [6] were used to determine the association between PHS resistance and *Qphs.hwwgr-4A*. Five sequence-tagged sites (STS) from three *Tamyb10* genes [25] were analyzed to determine QTL for GC. Amplification, separation and scoring of polymorphic chain reaction (PCR) products followed Zhang et al*.* [33].

The GWAS panel was also genotyped with the Wheat 9K and 90K SNP arrays [37, 38] at USDA-ARS Cereal Crops Research Unit (Fargo, ND). SNPs with less than 5 % minor allele frequency (MAF) or with more than 15 % missing data were removed. A total of 5,921 and 21,600 SNPs were scored from the 9K and 90K SNP arrays, respectively. Association analysis was initially conducted using the 9K genotypic data, and 28 non-redundant SNPs with *p* < 0.001 were then selected and pooled together with the 90K data. Totally, 21,628 SNPs were used for the final analysis. Also, one SNP in the promoter region and two SNPs in the coding region of the *TaPHS1* gene [3, 4] were analyzed using three Kompetitive Allele Specific PCR (KASP) assays. Sequences that harbored significant SNPs and SSR markers were searched against the W7984 reference sequence to estimate their putative chromosome positions.

### Population structure and kinship

Population structure was characterized by a set of 1500 SNPs that are evenly distributed on all the 21 wheat chromosomes using the admixture model in STRUCTURE 2.3.4 [39]. *K*-values ran from 2 to 20 with 10 iterations set for each *k*-value. The burn-in time and replication number were set at 2 × 10^5^ and 2 × 10^4^, respectively. For each trait, Bayesian information criterion (BIC) [40] was applied to determine the optimum number of subpopulations. Marker-based kinship was estimated to approximate the probability of two individuals being identical by descent through adjusting the average probability of identical in the state between random individuals [41]. Kinship was calculated with the same set of 1,500 SNPs used for structure analysis using SPAGeDi package [42].

### Statistical analysis and genome-wide association analysis

Best linear unbiased predictions (BLUPs) were calculated for each accession evaluated in the greenhouse and field experiments using the 'lme4' package in R 3.2.2 [43] with year and location as random effects in the model. Genome-wide association analysis was conducted using two models: the generalized linear model (GLM) with the Q matrix as fixed effects, and the mixed linear model (MLM) with a Q matrix as fixed effects and a kinship matrix as random effects. These two models were applied to each experiment for GC and PHS resistance, and model fitness was determined based on the BIC values. Association analysis of SNP data was conducted using the genome association and prediction integrated tool (GAPIT) implemented in R [44], and association analysis of SSR data was conducted using PROC MIXED procedure in SAS 9.3 (SAS Institute Inc., Cary, NC). A threshold of *p* < 0.001 was set to claim significant associations between SSR markers and the traits (GC and PHS resistance), and *p* < 0.0001 was set to claim significant associations between SNPs and the traits. Linkage disequilibrium and haplotype analyses of the significant SNPs were performed with HAPLOVIEW v.4.2 (http://www.broadinstitute.org/scientific-community/science/programs/medical-and-population-genetics/haploview/haploview).

### QTL analysis

A linkage map covering the 4A QTL region was constructed for the RIL population of Tutoumai A x Siyang 936 using KASP markers converted from significant SNPs from the association study, and previously mapped SSR markers in Liu et al. [45] and GBS-SNPs in Lin et al. [7] by JoinMap version 4.0 [46]. Recombination fractions were converted into centiMorgans (cM) using the Kosambi function [47]. Interval mapping (IM) using sprouting data from the 2005 and 2006 greenhouse experiments and their combined mean was performed using WinQTLCart 2.5 [48]. LOD thresholds to claim significant QTL for each dataset were determined from 1000 permutations [49].

## Results

### Phenotypic variations in grain color and pre-harvest sprouting

**Fig. 1:**
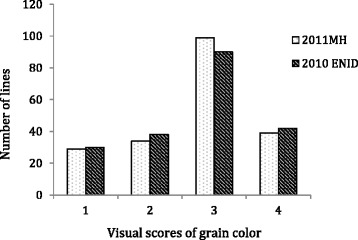
Frequency distribution of grain color (GC) scores evaluated using a 1 to 4 scale (white, light red, red and dark red) in the association mapping population. The seeds were harvested from the Manhattan 2011 greenhouse (2011MH) experiment and the Enid 2010 field (2010 ENID) experiment

Twenty-nine accessions were scored as white wheats, and 156 accessions as red wheats. The GC scores were highly consistent between greenhouse and field grown seeds (Fig. 1) with a high correlation coefficient of 0.87 (*P* < 0.0001), indicating a low genotype-by-environment interaction for GC.

**Fig. 2:**
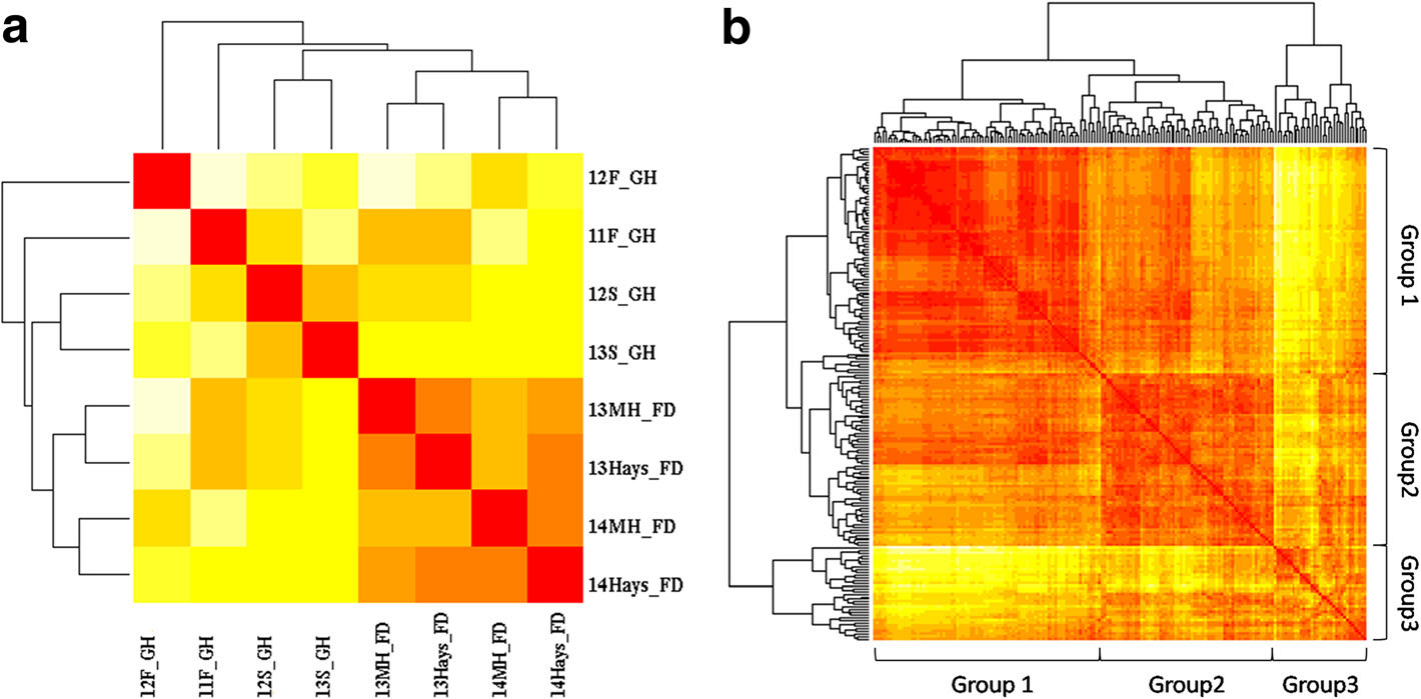
Heatmaps showing **a** the relationships of pre-harvest sprouting data among four greenhouse (GH) experiments conducted at Manhattan, KS in fall 2011(11F_GH), fall 2012(12F_GH), spring 2012(12S_GH), spring 2013(13S_GH) and four field experiments conducted at Manhattan in 2013 (13MH_FD) and 2014 (14MH_FD), and Hays in 2013 (14Hays_FD) and 2014 (14Hays_FD), and **b** the relationships and grouping of wheat accessions that were determined using the mean pre-harvest sprouting data collected from all four greenhouse and four field experiments. Similarity levels increase from light yellow (the lowest similarity) to dark red (the highest similarity)

Significant correlations for sprouting rates were observed among most of the eight experiments (Table 1). Cluster analysis showed high similarities in sprouting rates of accessions among all the field experiments, but significant differences between the field and the greenhouse experiments (Fig. 2a). The broad sense heritability across all eight experiments was high (0.83), with 0.62 in the greenhouse experiments and 0.92 in the field experiments. The population could be roughly divided into three subgroups (Fig. 2b), with average sprouting rates of 13.9 % in Group 1, 35.5 % in Group 2 and 60.3 % in Group 3, and average GC scores of 3.0 in Group 1, 2.6 in Group 2 and 1.7 in Group 3, indicating red wheats were more likely to have low sprouting rates. Most of the soft winter wheats were clustered to Group 1, as well as some hard white winter (HWW) wheat accessions from the Regional Germplasm Observation Nursery (RGON). The rest accessions from the RGON were mostly clustered to Group 2, whereas accessions from the Southern Regional Performance Nursery (SRPN) and the Northern Regional Performance Nursery (NRPN) were mainly clustered to Group 2 and Group 3.

**Table 1:** Pairwise correlation coefficients among germination rates from all eight experiments and best linear unbiased predictions (BLUP) of the all greenhouse experiments and all field experiments

Corr Coeff	2011F	2012S	2012F	2013S	2013_MH	2013_Hays	2014_MH	2014_Hays	GH_BLUP
2012S	0.238***								
2012F	0.468***	0.313***							
2013S	0.167*	0.543***	0.337***						
2013_MH	0.276***	0.171*	0.212**	0.153*					
2013_Hays	0.407***	0.227**	0.402***	0.237***	0.741***				
2014_MH	0.384***	0.312***	0.576***	0.242***	0.686***	0.721***			
2014_Hays	0.358***	0.228**	0.431***	0.266***	0.747***	0.755***	0.821***		
GH_BLUP	0.559***	0.718***	0.821***	0.710***	0.268***	0.437***	0.551***	0.448***	
Field_BLUP	0.399***	0.265***	0.463***	0.253***	0.869***	0.890***	0.909***	0.929***	0.484***

*p < 0.05; **p < 0.01, ***p < 0.001

### Genome-wide association studies on grain color

According to the BIC values, the mixed model with population structure and kinship fit the best for the GC trait, thus was applied in the following analysis. GWAS detected four significant QTL on chromosomes 1B, 3A, 3B and 3D, which were represented by the gene markers for *Tamyb10* genes (5 STS markers) and closely linked SSRs (6) and SNPs (12) (Table 2). Three major QTL for GC in the distal region of the long arms of group 3 chromosomes are significant for the data from both greenhouse and field experiments (Table 2). Among them, the QTL on 3DL, as indicated by significant markers *Tamyb10-D1* and 3 SNPs, showed the largest effect and explained up to 23.0 % of the phenotypic variance for GC. The QTL on chromosome 3BL that was characterized by seven SNPs, two gene makers for *Tamyb10-B1*, and one SSR was significant in both greenhouse and field experiments, and explained up to 19.2 % of the phenotypic variance. A QTL on 3AL showed a moderate effect on GC, and explained about 11.1 % phenotypic variance. QTL on chromosomes 1B was identified in both field and greenhouse experiments and explained up to 11.7 % of the phenotypic variances. Also, three SSRs, *Xwmc93*, *Xbarc145* and *Xbarc148*, were also significant for GC, but their positions cannot be determined because they were mapped to multiple chromosomes.

**Table 2:** Quantitative trait loci (QTL) identified for wheat grain color (GC) evaluated for the seeds harvested from the field experiment of Enid, OK, in,2010 (Enid2010) and from the greenhouse (GH) experiment conducted in Manhattan KS, 2011 (GH2011)

Chromosome	Marker name	Marker type	Chromosome Position (cM)^a^	Positive allele frequency	Enid2010		GH2011		Mean	
					*p*	*R* ^*2*^ (%)^b^	*p*	*R* ^*2*^ (%)	*p*	*R* ^*2*^ (%)
1B	*Ra_c35710_395*	90K	58.08	0.92	7.31E-06	9.9	2.19E-05	9.3	3.16E-06	10.8
1B	*RAC875_c1188_531*	90K	58.08	0.92	4.62E-06	10.4	8.06E-06	10.3	1.33E-06	11.7
3A	*Xwmc559-1*	SSR	107.20	0.94	1.25E-04	9.8	5.00E-04	10.6	1.57E-04	10.8
3A	*Tamyb10-A1-66*	STS	114.02	0.63	8.75E-06	7.5	1.86E-04	10.2	2.25E-05	9.4
3A	*Tamyb10-A1-74*	STS	114.02	0.61	3.12E-06	8.5	6.50E-05	11.1	7.32E-06	10.4
3B	*BS00040742_51*	90K	68.26	0.36	9.42E-06	9.7	-	-	2.82E-05	8.6
3B	*Tdurum_contig100004_204*	90K	-	0.38	4.81E-06	10.3	1.00E-05	10.1	7.54E-06	9.9
3B	*BS00025679_51*	90K	76.22	0.58	2.45E-05	8.7	1.88E-06	11.8	6.30E-06	10.1
3B	*Kukri_c60633_121*	90K	76.22	0.35	8.53E-06	9.8	2.35E-06	11.6	4.23E-06	10.5
3B	*Kukri_c60633_257*	90K	76.22	0.33	3.63E-05	8.3	5.35E-06	10.7	9.98E-06	9.6
3B	*Excalibur_rep_c97324_623*	90K	76.22	0.35	5.23E-06	10.3	1.64E-06	12.0	2.50E-06	11.0
3B	*Tamyb10-B1-1*	STS	77.36	0.26	2.26E-05	11.1	1.06E-05	11.0	7.46E-06	11.8
3B	*Tamyb10-B1-2*	STS	77.36	0.26	7.32E-07	11.1	8.21E-07	11.0	3.22E-07	11.8
3B	*Xbarc84*	SSR	80.77	0.31	1.89E-05	4.1	-	-	1.16E-04	6.4
3D	*GENE-1785_118*	90K	-	0.42	4.74E-06	10.4	2.42E-09	19.2	6.49E-08	14.8
3D	*D_GA8KES402JVT1Y_74*	90K	11.37	0.54	1.32E-07	14.0	3.31E-10	21.5	1.79E-09	18.7
3D	*BS00067163_51*	90K	92.34	0.52	5.36E-08	15.0	8.39E-11	23.2	7.49E-10	19.7
3D	*BS00063075_51*	90K	-	0.72	8.85E-05	7.5	5.03E-06	10.8	9.74E-06	9.7
3D	*Tamyb10-D1-93*	STS	-	0.56	4.31E-11	21.9	3.66E-13	17.5	6.51E-13	21.0
3D	*Xbarc376*	SSR	-	0.94	-	-	5.00E-04	24.6	7.00E-04	23.3
1A/1D	*Xwmc93*	SSR	-	0.37	-	-	6.10E-05	5.3	3.00E-04	7.2
1A/2D/3B	*Xbarc145*	SSR	-	0.11	-	-	6.62E-05	5.0	1.65E-04	6.7
1A/1D/3A/5B	*Xbarc148*	SSR	-	0.70	2.50E-07	16.6	1.03E-06	18.7	2.27E-07	18.4

^a^The marker positions in the chromosome based on W7984 reference map

^b^Phenotypic variance explained by a significant marker significantly related to grain color

**Fig. 3:**
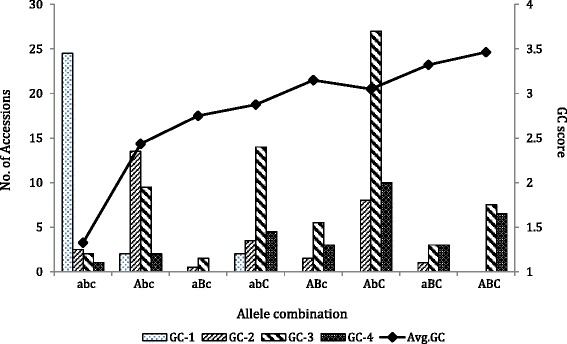
Distribution of grain color (GC) scores in the association mapping population predicted by *Tamyb10* gene markers. Six allele combinations of three GC genes on chromosomes A, B and D separated 185 accessions into eight genotypes. Lower case represents a white grain allele and upper case represents a red grain allele in each locus. The three letters in each genotype represent three gene loci in the chromosomes A, B and D, respectively, e.g, Abc indicates red allele on 3A and white alleles on 3B and 3D. GC scores used a 1-4 scale with 1 for white grain and 4 for red grain

The association mapping population can be classified into eight genotypic groups based on the allele combinations of the gene markers of *Tamyb10* genes on group 3 chromosomes. The average GC scores in each group tended to increase as the number of red color alleles increases. However, red wheat accessions T154, LA02-923, MO040192 and NC04-15533 do not contain the red alleles (abd) at any of the three loci, whereas white accessions KS05HW15-2 and OK06848W carry the red allele of *Tamyb10-A1* (Abd), and white accessions KS05HW136-3 and CO03W139 carry the red allele of *Tamyb10-D1* (abD) (Fig. 3), suggesting that other genes besides *Tamyb10* may also contribute to GC, or the markers for *Tamyb10* genes may not be diagnostic in some genetic backgrounds.

### Genome-wide association studies on pre-harvest sprouting resistance

Generalized linear model with Q matrix of k = 3 was selected for GWAS on PHS resistance based on BIC values. Twelve QTL on ten chromosomes were significant for PHS resistance in at least two experiments (Table 3; Additional file 3: Table S3). Among them, QTL on chromosome 3AS, 3AL, 3B and 4AL were the most frequently identified QTL for PHS resistance. The 3AS QTL was detected in the fall 2011 greenhouse experiment and all the four field experiments, and explained 9.5 % to 15.8 % of the phenotypic variances for PHS resistance. Significant markers included one SSR, *Xbarc57*, five SNPs from the SNP chips and one SNP developed from the *TaPHS1* gene sequence (Table 3; Additional file 3: Table S3), thus this QTL corresponds to *TaPHS1*. The 3AL QTL was identified by SNPs, SSR and the *Tamyb10-a1* gene marker in spring 2012 and all the field experiments, and explained 6.8 % to 12.1 % of the phenotypic variances. Thus this QTL corresponds to the *Tamyb10-a1* gene for GC on 3AL. The QTL on chromosome 4AL showed a wide range of effects among the experiments, and explained 9.9 % to 47.6 % phenotypic variance among the two greenhouse experiments (fall and spring 2012) and one field experiment (Manhattan, 2014). The most significant SNPs for *Qphs.hwwgr-4A* were *Ex_c66324_1151*, *wsnp_Ex_c13031_20625900* and *wsnp_Ex_rep_c66324_64493429. ZXQ118*, an EST in the 4A QTL region (Zhang et al., 2008), and the gene markers for *PM19A1* were also significant in the fall 2012 greenhouse experiment and the mean sprouting rates over all the greenhouse experiments, but explained much lower phenotypic variation than the previous three markers (Table 3; Additional file 3: Table S3). The QTL on chromosome 3B was significant in two greenhouse experiments (fall 2011 and spring 2012) and all field experiments, and explained 7.0 % to 12.3 % of the phenotypic variances for PHS resistance. Two QTL were identified on chromosome 3D with one at the distal end of the short arm (*Qphs.hwwgr-3DS*) and another at the distal end of the long arm (*Qphs.hwwgr-3DL*). *Qphs.hwwgr-3DS* was detected in the spring 2012 greenhouse, and two 2014 field experiments, and *Qphs.hwwgr-3DL* was significant in both field experiments in 2013. QTL identified on chromosome 7A was significant in the fall 2011 greenhouse and 2013 Manhattan field experiments and explained up to 13.5 % of the phenotypic variance.

**Table 3:** Representative markers for quantitative trait loci (QTL) of wheat pre-harvest sprouting resistance identified in at least two of the experiments using sprouting rates (%) evaluated in the fall 2011 (2011F), spring 2012 (2012S), fall 2012 (2012F) and spring 2013 (2013S) greenhouse experiments, the 2013 and 2014 Manhattan (2013MH and 2014MH) and 2013 and 2014 Hays (2013Hays and 2014Hays) field experiments, and using the best linear unbiased predictions (BLUP) of each accession from all the greenhouse (GH_BLUP) and field (Field_BLUP) experiments

Chromosome	Marker name	Marker type	Chromosome position (cM)^a^	Positive allele frequency	*p*	*R* ^*2*^ (%)^b^	Experiment
1A	*BS00011787_51*	90K	34.28	0.49	3.30E-05	9.2	2013MH
1A	*Kukri_c60564_136*	90K	50.20	0.93	6.54E-05	9.1	2011F
1D	*Ex_c6765_2118*	90K	48.90	0.83	5.74E-04	6.7	2013S
1D	*wsnp_Ku_c19622_29138795*	90K	-	0.67	3.38E-04	7.3	2013S
1D	*Xgwm337*	SSR	-	0.18	4.98E-04	17.8	2012S
					1.30E-04	13.3	2013S
					4.70E-04	13.5	GH_BLUP
2D	*BobWhite_c1477_315*	90K	-	0.33	9.48E-04	6.2	2013S
2D	*Xgwm539*	SSR	-	0.11	5.00E-04	11.7	2013MH
					6.00E-04	15.1	2014Hays
					3.38E-04	16.7	Field_BLUP
3AS	*wsnp_Ex_rep_c67702_66370241*	9K	9.12	0.78	4.42E-05	9.5	2011F
3AS	*wsnp_Ra_c2339_4506620*	9K	9.12	0.38	5.28E-05	8.7	2013MH
					5.66E-05	8.5	Field_BLUP
3AS	*KASP-TaPHS1.1*	STS	-	0.78	5.55E-05	9.3	2011F
3AS	*Xbarc57.2*	SSR	-	0.79	6.74E-06	14.0	2013MH
					6.56E-06	14.2	2013Hays
					9.46E-05	15.3	2014MH
					2.00E-04	13.7	2014Hays
					3.78E-06	15.9	Field_BLUP
3AL	*wsnp_Ku_c5359_9531713*	9K	L201.88	0.89	2.50E-04	7.6	2012S
3AL	*Tamyb10-A1-66*	STS	114.02	0.61	4.67E-07	12.1	2013MH
					1.82E-05	9.2	2013Hays
					3.00E-04	6.8	2014MH
					2.83E-05	8.6	2014Hays
					2.01E-06	10.9	Field_BLUP
3AL	*Xwmc559-1*	SSR	107.20	0.89	9.00E-04	10.4	2013Hays
3B	*wsnp_BE446087B_Ta_2_1*	9K	46.66	0.91	3.22E-05	9.3	2013Hays
3BL	*Xbarc77*	SSR	-	0.94	5.33E-04	12.3	2012S
					4.00E-04	7.6	2013MH
					2.00E-04	9.5	2014Hays
					4.82E-04	8.1	Field_BLUP
3BL	*Xgwm108*	SSR	-	0.81	9.00E-04	7.0	2013MH
					2.00E-04	7.5	2014MH
					5.68E-04	7.3	Field_BLUP
3BL	*Xgwm181*	SSR	-	0.92	3.00E-04	12.2	2011F
3DS	*BS00067117_51*	90K	-	0.47	8.26E-06	11.1	2014MH
					8.56E-05	7.4	2014Hays
3DS	*Kukri_c50527_241*	90K	-	0.35	8.46E-04	6.3	2012S
					2.97E-05	9.7	2014MH
3DS	*BobWhite_c3111_636*	90K	-	0.32	1.15E-05	10.8	2014MH
					8.88E-05	7.4	2014Hays
					7.09E-05	8.3	Field_BLUP
3DL	*BS00067163_51*	90K	92.34	0.52	7.79E-05	8.3	2013MH
3DL	*Tamyb10-D1-93*	STS	-	0.56	6.00E-04	5.7	2013MH
					3.00E-04	6.7	2013Hays
4A	*Ex_c66324_1151*	90K	76.97	0.42	3.67E-17	47.6	2012F
					2.37E-05	9.9	2014MH
					1.34E-12	31.5	GH_BLUP
4A	*wsnp_Ex_rep_c66324_64493429*	90K	76.97	0.43	1.51E-16	45.3	2012F
					2.60E-05	9.8	2014MH
					2.99E-12	30.4	GH_BLUP
4A	*Xbarc236*	SSR	92.92	0.23	7.41E-05	14.1	2012F
4A	*Xwmc757*	SSR	-	0.11	9.84E-04	11.9	2012S
5A	*BobWhite_c4004_61*	90K	35.36	0.83	2.62E-04	7.6	2013S
5A	*Excalibur_c54774_408*	90K	47.99	0.77	5.60E-04	6.7	2012S
6B.2	*wsnp_Ex_c19525_28494827*	90K	94.46	0.91	1.21E-05	10.2	2013MH
					8.19E-06	10.8	2013Hays
					2.81E-05	9.3	Field_BLUP
6B.2	*Excalibur_c15109_942*	90K	95.60	0.64	4.29E-04	7.0	2013S
6B	*Xgwm88*	SSR	-	0.91	9.55E-04	8.9	2012S
7A	*wsnp_Ex_c26509_35755018*	9K	69.63	0.95	1.41E-06	13.5	2011F
7A	*Xwmc603*	SSR	-	0.92	1.00E-04	25.4	2011F
7A	*Xgwm130*	SSR	-	0.94	5.00E-04	10.6	2013MH
1A/1D/3A/5B	*Xbarc148*	SSR	-	0.83	1.38E-08	22.8	2013MH
					3.38E-05	11.9	2013Hays
					2.22E-05	14.5	2014Hays
					3.43E-06	14.7	Field_BLUP
5ABD	*Xbarc232*	SSR	-	0.92	6.00E-04	10.3	2013MH
					4.40E-05	12.6	2014MH
					3.97E-05	13.6	2014Hays
					3.80E-04	10.9	Field_BLUP

^a^The positions of markers on W7984 reference sequence

^b^Phenotypic variance explained by a significant marker significantly related to pre-harvest sprouting resistance

Some QTL were only significant in a single environment. For example, the two QTL identified on chromosomes 2B, *Qphs.hwwgr-2B.1* and *Qphs.hwwgr-2B.2*, were associated with PHS resistance each in one greenhouse experiment (spring 2013 and 2012, respectively), and the *Qgc.hwwgr-6B.1* was identified only in one field experiment (Manhattan, 2013) (Table 4). Therefore, these QTL may be more sensitive to environmental conditions.

**Table 4:** Quantitative trait loci (QTL) for pre-harvest sprouting resistance identified in only one of the experiments conducted in the spring 2012 (2012S) and spring 2013 (2013S) greenhouse experiments and the 2013 Manhattan field experiment (2013MH)

Chromosome	Marker name	Marker type	Chromosome position (cM)^a^	Positive allele frequency	*p*	*R* ^*2*^ (%)^b^	Experiment
2B.1	*Excalibur_c1787_1199*	90K	7.97	0.81	2.97E-04	7.4	2013S
2B.1	*BS00044806_51*	90K	10.24	0.72	4.98E-04	6.9	2013S
2B.1	*Tdurum_contig51145_476*	90K	10.24	0.76	3.65E-04	7.2	2013S
2B.1	*BS00022203_51*	90K	-	0.17	7.08E-04	6.5	2013S
2B.1	*Excalibur_c3524_318*	90K	-	0.81	7.05E-04	6.5	2013S
2B.1	*Kukri_c16758_443*	90K	10.24	0.73	1.31E-04	8.3	2013S
2B.1	*wsnp_JD_c3288_4296662*	9K	10.24	0.77	7.57E-04	6.4	2013S
2B.1	*BS00065556_51*	90K	-	0.77	2.56E-04	7.6	2013S
2B.2	*wsnp_Ex_c13865_21720466*	9K	83.07	0.42	6.42E-04	6.6	2012S
2B.2	*wsnp_RFL_Contig3273_3319580*	90K	83.07	0.41	7.30E-04	6.4	2012S
2B.2	*RAC875_c26697_589*	90K	83.07	0.35	4.27E-04	7.0	2012S
2B.2	*Tdurum_contig28795_322*	90K	-	0.41	7.63E-04	6.4	2012S
6B.1	*RAC875_c23251_624*	90K	43.28	0.89	2.59E-05	9.4	2013MH
6B.1	*BS00066799_51*	90K	43.28	0.92	7.13E-05	8.3	2013MH
6B.1	*CAP8_c1361_367*	90K	43.28	0.89	2.59E-05	9.4	2013MH

^a^The positions of markers on W7984 reference sequence

^b^Phenotypic variance explained by a marker that was significantly associated with pre-harvest sprouting resistance

### Relationships between grain color and pre-harvest sprouting resistance

Analysis of variance (ANOVA) was conducted by taking CG as the explanatory variable and PHS resistance as the response variable, and it showed that GC had significant effects on PHS resistance in all the field experiments (*P* < 0.0001), but not in any of the greenhouse experiments (Table 5). White wheat had significantly higher sprouting rates than red wheats (*P* < 0.0001) in the field experiments, but the difference was not significant between red-grained accessions with different color scores (data not shown).

**Table 5:** Effect of grain color (GC) that was evaluated in the field at Enid, OK in 2010 (2010Enid) and the greenhouse at Manhattan KS 2011 (2011F_GH) on pre-harvest sprouting (PHS) resistance evaluated in four greenhouse experiments (GH_experiments) conducted in Manhattan, KS, and four field experiments conducted in Manhattan (MH) and Hays, KS in 2013 and 2014, respectively

Experiments	2010Enid (GC)		2011F_GH (GC)	
	*p*	*R* ^*2*^ (%)^a^	*p*	*R* ^*2*^ (%)^a^
GH_experiments (PHS)	NS	-	NS	-
2013MH (PHS)	<2e-16	43.7	<2e-16	44.5
2013Hays (PHS)	<2e-16	42.9	<2e-16	43.6
2014MH (PHS)	1.27E-10	26.3	3.13E-11	27.5
2014Hays (PHS)	1.13E-15	35.6	3.41E-15	34.8
Field_BLUP ^b^(PHS)	<2e-16	43.9	<2e-16	44.1

^a^Phenotypic variance explained by grain color in each PHS experiment, which is derived from the analysis of variance (ANOVA) where grain color (GC) was used as the explanatory variable and pre-harvest sprouting (PHS) resistance as the response variable

^b^Field_BLUP = Best Linear Unbiased Predictions calculated from all four field experiment

Common QTL for GC and PHS resistance were identified on the long arms of chromosomes 3A and 3D (Table 6), but not on 3BL. The QTL on chromosome 3AL, identified by *Tamyb10-A1*, was significant for GC in both field and greenhouse experiments and for PHS resistance in all the field experiments. For the QTL on chromosome 3D as represented by *Tamyb10-D1*, one SNP was significant for GC in both experiments and also for PHS resistance in the 2013 field experiments at both Manhattan and Hays. Unlike the 3A and 3D QTL, QTL on chromosome 3B, represented by the *Tamyb10-B1* as well as seven linked SNPs and one SSR, was significant for only GC, not PHS resistance in any experiments. Therefore, *Tamyb10-A1* and *Tamyb10-D1*, but not *Tamyb10-B1*, were very likely to have pleiotropic effects on PHS resistance under the field conditions.

**Table 6:** Common Quantitative trait loci (QTL) identified for grain color evaluated in 2010 field (2010Enid) and 2011 greenhouse (GH) experiments and pre-harvest sprouting resistance evaluated in Manhattan (MH) and Hays in 2013 and 2014 experiments, respectively

			Grain color						PHS resistance									
Chromosome	Marker name	Chromosome position (cM)^a^	2010Enid		2011GH		Mean		2013MH		2013Hays		2014MH		2014Hays		Field_BLUP	
			*p*	*R* ^*2*^ (%)^b^	*p*	*R* ^*2*^ (%)^b^	*p*	*R* ^*2*^ (%)^b^	*p*	*R* ^*2*^ (%)^c^	*p*	*R* ^*2*^ (%)^c^	*p*	*R* ^*2*^ (%)^c^	*p*	*R* ^*2*^ (%)^c^	*p*	*R* ^*2*^ (%)^c^
3AL	*Xwmc559-1*	107.20	1.25E-04	9.8	5.00E-04	10.6	1.57E-04	10.8	-	-	9.00E-04	10.4	-	-	-	-	-	-
3AL	*Tamyb10-A1-66*	114.02	8.75E-06	7.5	1.86E-04	10.2	2.25E-05	9.4	4.67E-07	12.1	1.82E-05	9.2	3.00E-04	6.8	2.83E-05	8.6	2.01E-06	10.9
3AL	*Tamyb10-A1-74*	114.02	3.12E-06	8.5	6.50E-05	11.1	7.32E-06	10.4	8.07E-07	11.6	3.47E-05	8.6	-	-	1.16E-04	7.3	1.12E-05	9.4
3DL	*BS00067163_51*	92.34	5.36E-08	15.0	8.39E-11	23.2	7.49E-10	19.7	7.79E-05	8.3	-	-	-	-	-	-	-	-
3DL	*Tamyb10-D1-93*	-	4.31E-11	21.9	3.66E-13	17.5	6.51E-13	21.0	6.00E-04	5.7	3.00E-04	6.7	-	-	-	-	-	-
1A/1D/3A/5B	*Xbarc148*	-	2.50E-07	16.6	1.03E-06	18.7	2.27E-07	18.4	1.38E-08	22.8	3.38E-05	11.9	-	-	2.22E-05	14.5	3.43E-06	14.7

^a^The marker positions in a chromosome based on W7984 reference map

^b^Phenotypic variance explained by a marker that is significantly associated with grain color

^c^Phenotypic variance explained by a marker that is significantly associated with pre-harvest sprouting resistance

### Validation of the significant SNPs for the 4A QTL in a bi-parental population

**Fig. 4:**
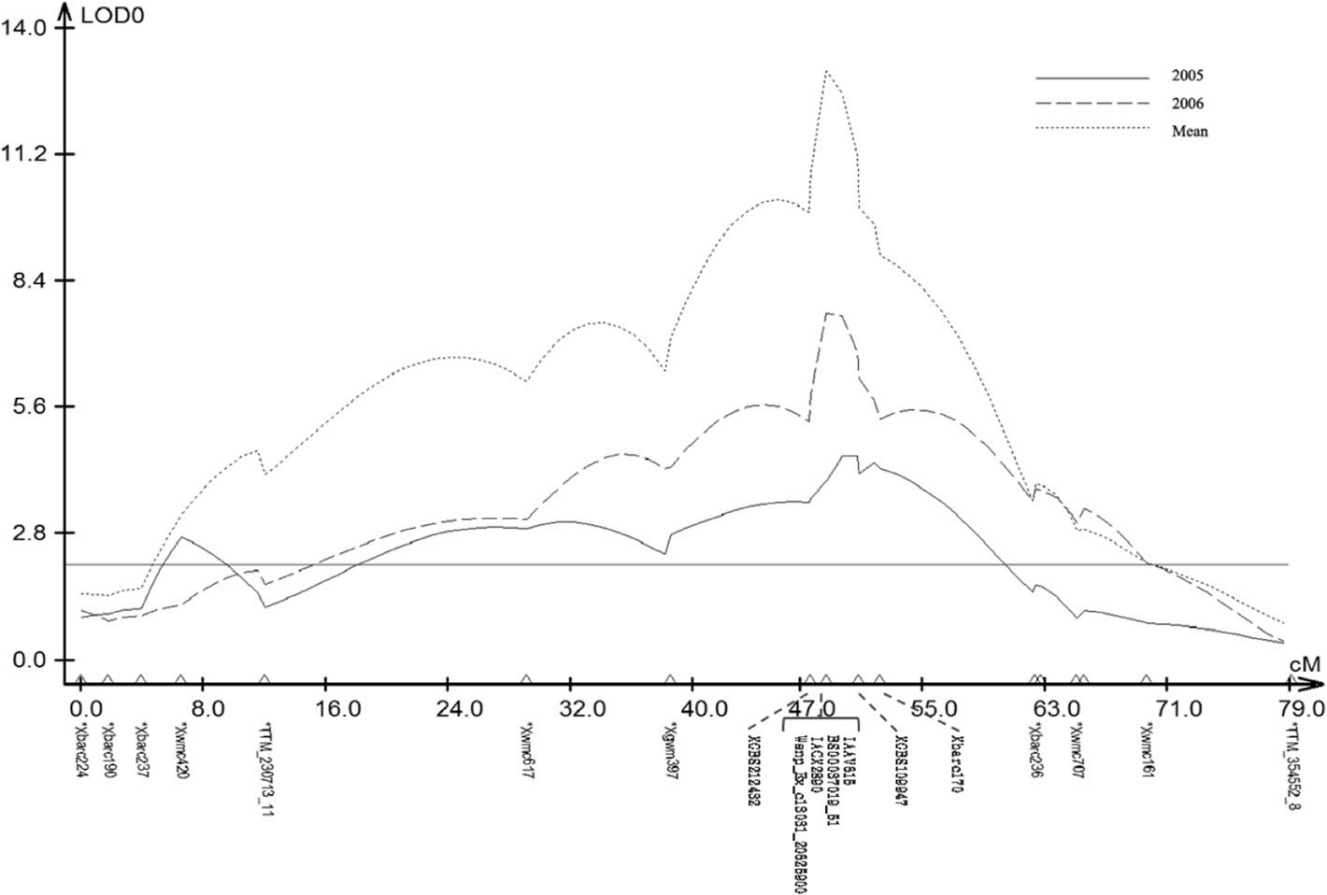
Interval mapping (IM) of a quantitative trait locus (QTL) for pre-harvest sprouting (PHS) resistance on chromosome 4A using SSRs, GBS-SNPs and SNPs identified from genome-wide association study (GWAS). The line parallel to the X-axis is the threshold line for the significant LOD value of 2.42 (*P* < 0.05). Genetic distances are in centiMorgans (cM)

Seventeen KASP assays were designed based on the sequences of the significant SNPs identified in the 4A QTL region for PHS resistance. Four of the KASP markers (Additional file 4: Table S4) showed co-segregation among the F_6_ RILs of 'Tutoumai A' × 'Siyang 936', and were mapped between the two previously reported flanking GBS SNPs (*GBS212432*, *GBS109947*) for the QTL [7] at 1.02 cM to *GBS212432* and 2.10 cM to *GBS109947* (Fig. 4). These four SNPs showed the highest LOD scores in all experiments, and explained up to 31.76 % of the phenotypic variance in the population.

### Linkage disequilibrium

**Fig. 5:**
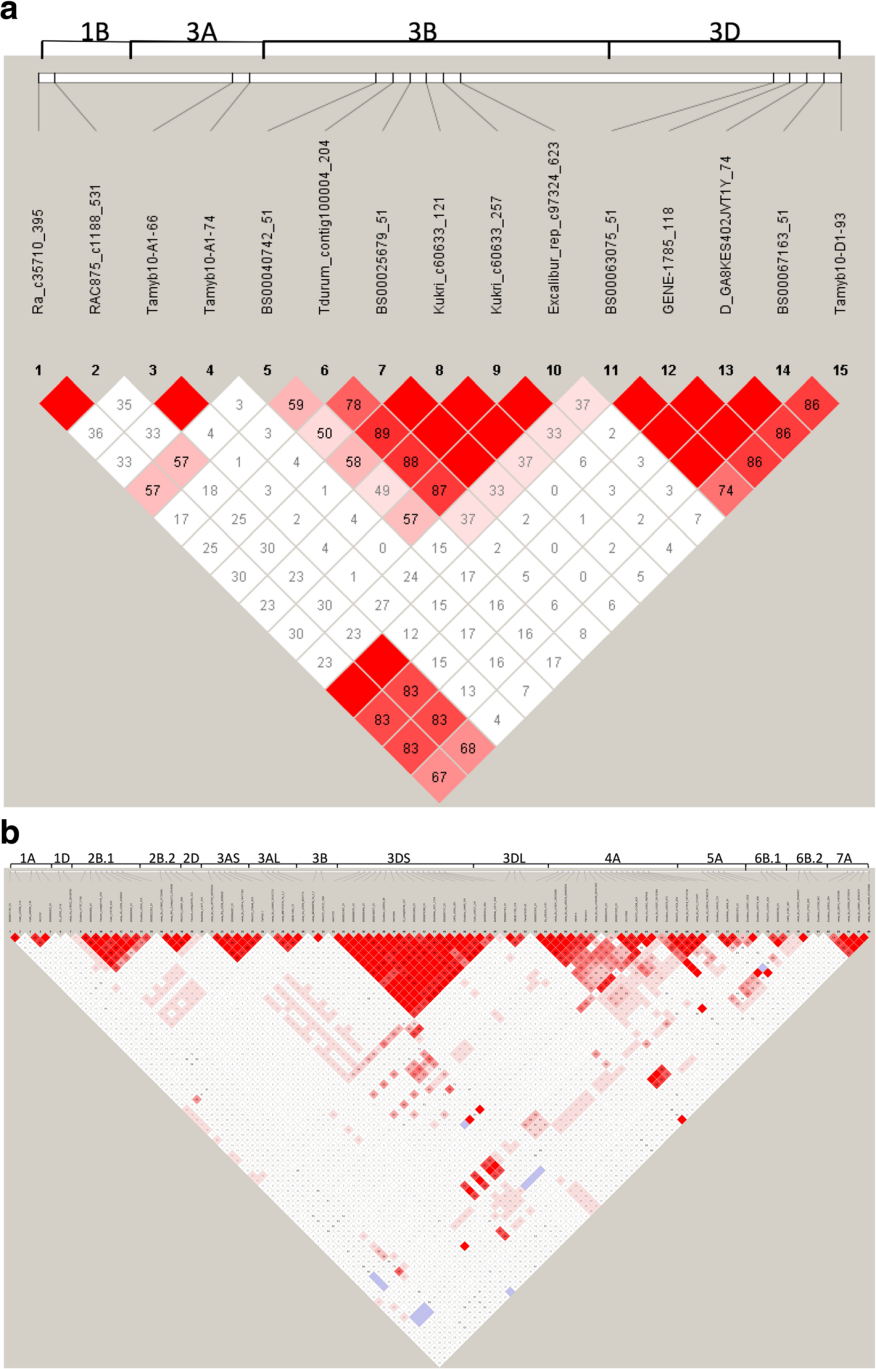
LD plots of SNP markers that showed significantly association with GC (**a**) and pre-harvest sprouting (PHS) resistance (**b**). The chromosome numbers are labeled above the chromosome maps (the long white bar) and marker names are labeled between the LD plot and chromosome maps

Linkage disequilibrium (LD) parameter *r* ^2^ was calculated to determine the linkage relationship between SNPs from different QTL and link the markers with unknown positions to known QTL. LD was calculated for the 125 SNPs that were significantly linked to nine PHS resistance QTL in at least two experiments. Strong LD was detected for SNPs within each PHS resistance QTL region, but not between different QTL (Fig. 5b), indicating that those QTL for PHS resistance were independent. Pair-wise *r* ^2^ values were also estimated for the 17 SNPs that were tightly linked to the five GC QTL. Similarly, strong LD was not detected among the SNPs linked to GC QTL in group 3 chromosomes (Fig. 5a).

Genetic positions of most significant SNPs for GC on chromosome 3D and *Tamyb10-D1* could not be determined using the W7984 reference sequence, and SNPs significantly related to GC on chromosome 3D, *D_GA8KES402JVT1Y_74* and *BS00067163_51*, were far apart from each other on the chromosome 3D. However, LD analysis suggested that these SNPs were tightly linked to *Tamyb10-D1*, and thus they linked to the same 3D QTL for GC (Fig. 5a).

## Discussion

### QTL for grain color

Wheat GC has been a classic example for dissection of a quantitative trait [50] and three genes on wheat chromosomes group 3 have long been proposed as the genes controlling wheat GC. Several previous studies have mapped the three genes as major QTL as well as some minor QTL on chromosomes 2B, 2D, 5A and 6B for GC [1, 25, 51]. Being the first association study for wheat GC, we not only validated the effects of these three GC genes, *Tamyb10-A1*, *Tamyb10-B1* and *Tamyb10-D1*, on the long arms of chromosomes group 3, but also identified a new QTL on the chromosome 1B for GC, suggesting that QTL on other chromosomes than these well-known QTL on chromosomes group 3 may also play a role in regulating GC in some wheat germplasm lines.

Groos et al. [1] mapped all group 3 QTL in a bi-parental population, but they did not discuss their effects of each QTL. In this study, a diverse association panel makes it possible to compare the effects of all the three QTL. Among the three genes on the chromosome group 3, *Tamyb10-D1* had the largest effect on GC (R^2^ = 0.24) and *Tamyb10-A1* the smallest (R^2^ = 0.11) in the association mapping panel whereas their minor allele frequencies (MAF) were similar (Table 2), indicating that the large effect of *Tamyb10-D1* was not due to a higher MAF than other two genes. On the other hand, one single gene changed GC from white to red, and adding one or two additional GC genes only slightly increased redness (Fig. 3). Besides, QTL on chromosome 1B also contribute to GC, which was not reported previously, thus it is likely a new QTL for GC. That the red allele of the 1B QTL presents in the four red wheat accessions that do not carry the red alleles (abd) at any of the three *Tamyb10* genes supports this assumption. Therefore, when breeding for white wheat cultivars, breeders not only need to remove the three *Tamyb10* genes, but also should watch for other genes that may contribute to GC.

In this study, wheat GC was visually scored after increasing color intensities using sodium hydroxide solution. High repeatability in GC between the greenhouse and field experiments (Fig. 1) indicates that the GC scoring method used in the experiments is highly repeatable. All of the four QTL identified for GC were detected in both experiments, which provided genetic evidence that QTL for GC are relatively stable across environments.

### QTL for pre-harvest sprouting resistance

QTL for PHS resistance have been mapped on almost all wheat chromosomes in previous bi-parental mapping studies. Although association studies on PHS resistance have been conducted using several types of markers [11, 30], the current study is the first report to use high density SNPs for GWAS on PHS resistance. We identified 12 QTL that were significance in at least two experiments.

For the QTL on 3AS, the causal gene (*TaPHS1*) has been cloned [3, 4]. One of the reported functional SNPs in the coding region [4] was significant in one greenhouse (fall 2011), whereas the functional SNP in the promoter region [3] was not significant in any of the experiments (Table 3; Additional file 3: Table S3). However, the most significant markers linked to the 3AS PHS resistance QTL were not the functional SNPs (Additional file 3: Table S3), which was probably due to environmental effects on phenotyping [3]. We had similar result for markers in the 4A QTL region. Only one of the candidate gene markers of *PM19A1* was significantly associated with PHS resistance in the fall 2012 experiment, although the 4A QTL showed an extremely large effect on PHS in that experiment (Table 3; Additional file 3: Table S3). This was probably due to the fact that the gene expression was affected by environments or the gene markers are not diagnostic.

The QTL identified at the distal end of chromosome 3DS was not reported previously. LD analysis indicated that *Qgc.hwwgr-3DS* is different but could be a homeologue of *Qgc.hwwgr-3AS* (Fig. 5b). For the QTL on chromosome 3B, the sequences of the linked SSR markers are not found in the W7984 reference sequence, thus we cannot determine whether or not the significant SSR markers and SNPs on 3B linked to the same QTL. Similarly, we cannot determine the QTL positions on chromosome 7A.

QTL identified on chromosome 1A could be the same QTL reported by Knox et al. [52] in durum because *Xwmc183* was located near the QTL region mapped in our study based on the W7984 reference sequence. The QTL on chromosome 2D is the same QTL as *QPhs.ccsu-2D.4* [53] because of the common SSR *Xgwm539*. However, we cannot determine whether the QTL that were identified on chromosomes 1D, 5A, 5B, 6A and 6B were the same QTL reported in previous studies [1, 9, 30, 51, 54] due to the lack of common markers.

### Variation of PHS resistance across environments

PHS is a complicated trait affected by many factors, including seed dormancy (SD) [15, 55–57], GC [1, 58], spike morphology, as well as environmental factors such as temperature, moisture and photoperiod after flowering [59, 60]. In the current study, PHS resistance of the tested accessions and QTL effects varied across environments with more variation observed among the greenhouse experiments than that among the field experiments (Fig. 2a). A total of four greenhouse experiments were conducted in the fall greenhouse cycles of 2011 and 2012 with the harvest time in winter, and the spring cycles of 2012 and 2013 with the harvest time in summer. The two seasons were highly different in growing and post-harvesting temperatures, which has been shown to influence PHS resistance [3, 6]. Meanwhile, in the field experiments at Manhattan and Hays, dry hot winds shortened maturity period, which greatly reduced environment effects on wheat PHS resistance. Therefore, PHS resistance was similar in the four field experiments.

*Qphs.hwwgr-3AS* and *Qphs.hwwgr-4A* were the major QTL for PHS resistance, and most frequently identified in all experiments. However, *Qphs.hwwgr-3AS* was detected more frequently in the field experiments, while *Qphs.hwwgr-4A* was detected more frequently in the greenhouse conditions (Table 3; Additional file 3: Table S3), which might be due to high temperatures in field conditions during late grain maturation that suppressed the expression of *Qphs.hwwgr-4A* [6].

According to the heat map derived from individual PHS ratings across all the experiments, the population can be roughly divided into three clusters (Fig. 2b). Most of the soft winter wheats had low germination rates, and were clustered to Group 1. Wheat cultivars from RGON were mostly clustered to Group 1 and Group 2, whereas accessions in SRPN and NRPN showed higher germination rates, and were mainly clustered to Group 2 and Group 3. These results indicated that the soft winter wheat accessions grown in the humid climate during harvest season had a higher selective pressure on PHS resistance than the hard winter wheat accessions from the Great Plains that grown under relatively drier climate.

### Validation of the markers for the QTL on 4A

In this study, a RIL population from ‘Tutoumai A’ x ‘Siyang 936’ was used to validate the position of significant SNPs for 4A PHS resistance QTL. Four polymorphic SNPs from GWAS were successfully mapped to the QTL region, and they are more closely linked to PHS resistance than previously reported flanking markers, *GBS212432* and *GBS109947*, for this QTL [7]. This result indicates that GWAS provides more power to increase marker density and mapping resolution, whereas bi-parental populations can further validate the positions of new markers. Barrero et al. [6] proposed *PM19A1* and *PM19A2* as the candidate genes for the 4A QTL and identified causal deletions in *PM19A1* and *PM19A2*. We analyzed the markers developed based on the causal variation in Tutoumai A and Siyang 936, but did not find any polymorphism between the two parents. Therefore, a different gene or different causal SNP in the gene may control the PHS resistance of 4A QTL in this population, which was also supported by the results from the GWAS that the candidate gene markers contributed much lower phenotypic variation for PHS resistance than three other SNP markers (*Ex_c66324_1151*, *wsnp_Ex_c13031_20625900*, *wsnp_Ex_rep_c66324_64493429*) (Table 3; Additional file 3: Table S3).

### Effect of grain color QTL on pre-harvest sprouting resistance

GC has been considered as an important factor for PHS resistance, and previous studies showed that seed dormancy level of a white-grained wheat line was improved by the introgression of an R gene [16]. In the current study, GC explained 26 % to 44 % of the phenotypic variance for PHS resistance, and *Tamyb10-A1* and *Tamyb10-D1* showed significant effects on both GC and PHS resistance, which agree with a previous study [1]. *Tamyb10* genes encode R2R3-type MBY transcription factors, which regulate the accumulation of PA in the biosynthesis pathways [25]. Therefore, it is possible that these transcription factors showed pleiotropic effects by regulating more than one metabolism pathway, and had effects on improving wheat PHS resistance. However, the GC gene on 3BL, *Tamyb10-B1*, did not show any effect on PHS resistance in this study (Table 2; Table 6).

In this study, GC was significantly related to PHS resistance in field experiments, but had barely any effect in the greenhouse experiments (Table 5). Also, the *Tamyb10-A1* gene affected PHS resistance in all of the four field experiments, and the *Tamyb10-D1* gene only affected PHS resistance in the 2013 experiments. Such results suggested that environmental factors could be important triggers of pleiotropic effects of the GC genes on PHS resistance. That *Tamyb10-B1* did not show any effect on PHS resistance might be due to the field environments of this study that could not trigger the expression of pleiotropic effect of the gene.

Although some GC genes contributed to wheat PHS resistance, many QTL for PHS resistance did not affect GC. Therefore, some red wheats can be highly susceptible to PHS, while some white wheats can be highly resistant [61, 62]. Breeding for PHS resistance, attention should be paid to these QTL with a major effect on PHS in most environments without a pleiotropic effect on GC, such as these on 3AS and 4AL. Pyramiding several of these genes in one cultivar should be able to avoid PHS damage in U.S. HWW.

## Conclusions

Using genome-wide association mapping, we identified four QTL for GC and 12 QTL for PHS resistance using 9K and 90K wheat SNP arrays. Besides three *Tamyb10* genes cloned from group 3 chromosomes showed significant effects on GC, a new QTL on chromosome 1B also contributed to GC. Among them, *Tamby10-A1* and *Tamyb10-D1* showed a pleiotropic effect on PHS resistance under field conditions. Several other QTL that did not affect GC trait, especially the QTL on chromosomes 3AS and 4AL, showed significant effects on PHS resistance, thus they can be pyramided to improve PHS resistance in white wheat cultivars.

## Additional files

Additional file 1:
**Table S1.** Pedigree, sources, origins and preharvest sprouting phenotypic data of 185 accessions in the genome-wide association study panel. (XLSX 121 kb)
Additional file 2:
**Table S2.** Environmental statistics of greenhouse and field conditions in Manhattan and Hays (XLSX 44 kb)
Additional file 3:
**Table S3.** Quantitative trait loci identified at least twice among eight experiments using the association mapping population and the Best Linear Unbiased Prediction (BLUP) of greenhouse and field data of wheat sprouting rates in a spike. (XLSX 59 kb)
Additional file 4:
**Table S4.** Kompetitive Allele Specific PCR assays developed from significant SNPs for the 4A pre-harvest sprouting resistance quantitative trait locus. (XLSX 33 kb)

## Abbreviations

PHS: Pre-harvest sprouting
GC: Grain color
QTL: Quantitative trait loci
GWAS: Genome-wide association study
SNP: Single nucleotide polymorphism
LD: Linkage disequilibrium
RIL: Recombinant inbred line
STS: Sequence-tagged site
PCR: Polymorphic chain reaction
MAF: Minor allele frequency
KASP: Kompetitive Allele Specific PCR
BIC: Bayesian information criterion
BLUP: Best linear unbiased prediction
GAPIT: Genome association and prediction integrated tool
IM: Interval mapping
GBS: Genotyping-by-sequencing
RGON: Regional Germplasm Observation Nursery
SRPN: Southern Regional Performance Nursery
NRPN: Northern Regional Performance Nursery
SD: Seed dormancy
